# 5-Nitro-1-benzofuran-2(3*H*)-one

**DOI:** 10.1107/S1600536812025093

**Published:** 2012-06-13

**Authors:** Xin-Yi Han, Ya-Bin Shi, Hong Shen, Shu-Yuan Bai, Hai-Bo Wang

**Affiliations:** a College of Science, Nanjing University of Technology, Xinmofan Road No. 5 Nanjing, Nanjing 210009, People’s Republic of China; b College of Food Science and Light Industry, Nanjing University of Technology, Xinmofan Road No. 5 Nanjing, Nanjing 210009, People’s Republic of China

## Abstract

In the crystal structure of the title compound, C_8_H_5_NO_4_, essentially planar mol­ecules [largest deviation from the least-squares plane = 0.030 (2) Å] form stacks along the *a*-axis direction. Intercentroid separations between overlapping benzene rings within the stack are 3.6594 (12) Å and 3.8131 (12) Å. Mol­ecules from neighboring stacks are linked by weak C—H⋯O hydrogen bonds into inversion dimers.

## Related literature

The title compound is an inter­mediate in the synthesis of the drug dronedarone [systematic (name: *N*-(2-butyl-3-(p-(3-(di­butyl­amino)­prop­oxy)benzo­yl)-5-benzofuran­yl)methane­sulf­on­amide, which has been used in the treatment of atrial fibrillation and atrial flutter. For applications of the title compound in drug discovery, see: Katritzky *et al.* (1992 ▶). For the synthetic procedure, see: Munoz-Muniz & Juaristi (2003 ▶). For standard bond-length data, see: Allen *et al.* (1987 ▶).
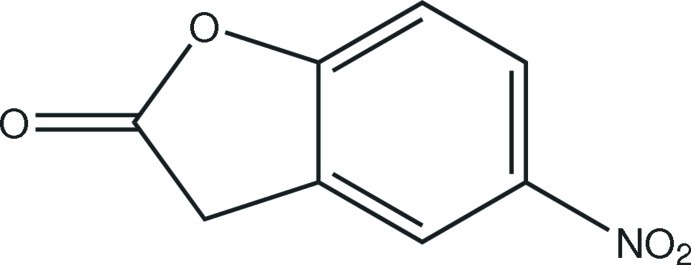


## Experimental

### 

#### Crystal data

C_8_H_5_NO_4_
*M* *_r_* = 179.13
Monoclinic, 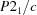

*a* = 7.4510 (15) Å
*b* = 8.9150 (18) Å
*c* = 11.249 (2) Å
β = 93.45 (3)°
*V* = 745.9 (3) Å^3^
*Z* = 4
Mo *K*α radiation
μ = 0.13 mm^−1^
*T* = 293 K
0.30 × 0.20 × 0.10 mm

#### Data collection

Enraf–Nonius CAD-4 diffractometer
Absorption correction: ψ scan (North *et al.*, 1968 ▶) *T*_min_ = 0.962, *T*_max_ = 0.987
2234 measured reflections
1362 independent reflections
1063 reflections with *I* > 2σ(*I*)
*R*_int_ = 0.039
3 standard reflections every 200 reflections intensity decay: 1%

#### Refinement

*R*[*F* ^2^ > 2σ(*F* ^2^)] = 0.046
*wR*(*F* ^2^) = 0.146
*S* = 1.00
1362 reflections
119 parameters
H-atom parameters constrained
Δρ_max_ = 0.22 e Å^−3^
Δρ_min_ = −0.20 e Å^−3^

### 

Data collection: *CAD-4 EXPRESS* (Enraf–Nonius, 1993 ▶); cell refinement: *CAD-4 EXPRESS*; data reduction: *XCAD4* (Harms & Wocadlo, 1996 ▶); program(s) used to solve structure: *SHELXS97* (Sheldrick, 2008 ▶); program(s) used to refine structure: *SHELXL97* (Sheldrick, 2008 ▶); molecular graphics: *SHELXTL* (Sheldrick, 2008 ▶); software used to prepare material for publication: *PLATON* (Spek, 2009 ▶).

## Supplementary Material

Crystal structure: contains datablock(s) global, I. DOI: 10.1107/S1600536812025093/yk2051sup1.cif

Structure factors: contains datablock(s) I. DOI: 10.1107/S1600536812025093/yk2051Isup2.hkl

Supplementary material file. DOI: 10.1107/S1600536812025093/yk2051Isup3.cml

Additional supplementary materials:  crystallographic information; 3D view; checkCIF report

## Figures and Tables

**Table 1:** Hydrogen-bond geometry (Å, °)

*D*—H⋯*A*	*D*—H	H⋯*A*	*D*⋯*A*	*D*—H⋯*A*
C3—H3*A*⋯O2^i^	0.97	2.56	3.334 (3)	137

Symmetry code: (i) 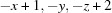.

**Fig. 1.**
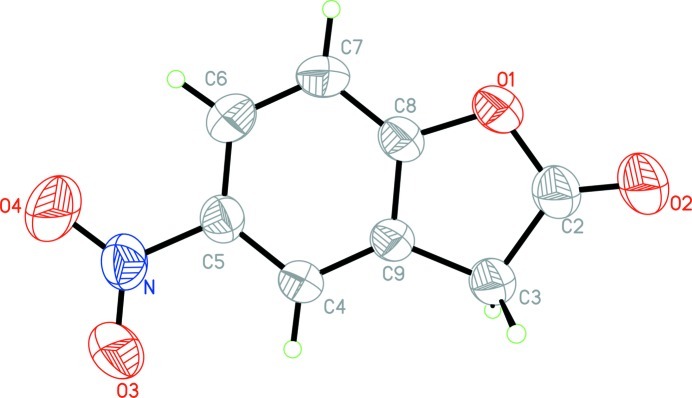
The molecular structure of the title compound, with the atom-numbering scheme and displacement ellipsoids drawn at the 50% probability level.

**Fig. 2.**
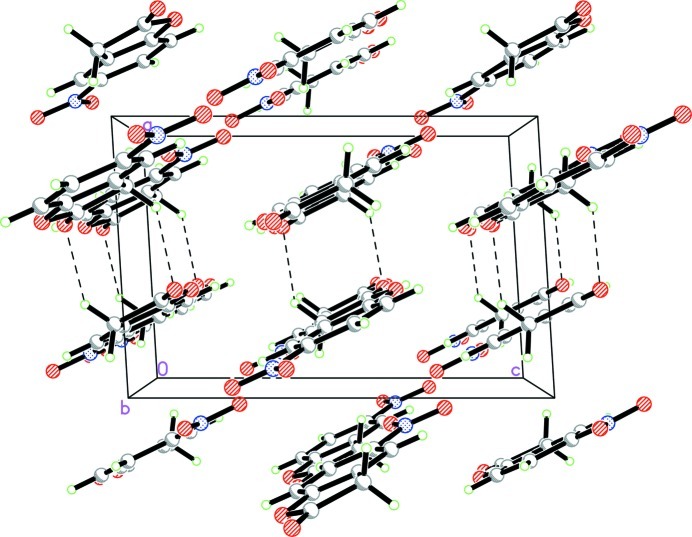
Packing diagram of the title compound. Hydrogen bonds are shown as dashed lines.

## supplementary crystallographic information

### Comment

The title compound, C_8_H_5_NO_4_ (Fig. 1), is an important pharmaceutical intermediate (Munoz-Muniz & Juaristi, 2003). We report here its crystal structure. All bond lengths and angles lie within the expected ranges (Allen *et al.*, 1987). In the crystal structure, the molecules are joined by π-π interactions and weak C-H···O hydrogen bonds (Fig. 2).

### Experimental

The title compound, 5-nitro-1-benzofuran-2(3H)-one, was prepared by the literature method (Munoz-Muniz & Juaristi, 2003). To a 100 mL flask provided with Dean–Stark tramp and magnetic stirrer was added 2-hydroxyphenyl- acetic acid (4.4 g, 29 mmol) in 60 mL of toluene and catalytic amounts of p-TsOH. The mixture was refluxed for 4 h with removal of water and then the residual solvent was removed at reduced pressure to give 3H-benzofuran-2-one in quantitative yield (3.9 g), mp 325K. Then, a mixture of 65% nitric acid (4 ml) and glacial acetic acid (4 ml) was added dropwise to a solution of 3H-benzofuran-2-one (3.9 g) in acetic anhydride (25 ml) while the temperature was maintained below 293K. The mixture was stirred and refluxed for 1 hour and decomposed with ice and sulfuric acid. The precipitate was filtered off. Pure 5-nitro-1-benzofuran-2(3H)-one was obtained by recrystallisation from ethyl acetate, yield 70%. Crystals suitable for X-ray analysis were obtained by slow evaporation of a methanol solution.

### Refinement

All H atoms were positioned geometrically, with C—H = 0.93, 0.98 and 0.96 Å for aromatic, methine and methyl H, respectively, and constrained to ride on their parent atoms, with U_iso_(H) = *x*U_eq_(C,N), where *x* = 1.5 for methyl H and *x* = 1.2 for all other H atoms.

### Crystal data

**Table:**

C_8_H_5_NO_4_	*F*(000) = 368
*M**_r_* = 179.13	*D*_x_ = 1.595 Mg m^−^^3^
Monoclinic, *P*2_1_/*c*	Melting point: 453 K
Hall symbol: -P 2ybc	Mo *K*α radiation, λ = 0.71073 Å
*a* = 7.4510 (15) Å	Cell parameters from 25 reflections
*b* = 8.9150 (18) Å	θ = 10–14°
*c* = 11.249 (2) Å	µ = 0.13 mm^−^^1^
β = 93.45 (3)°	*T* = 293 K
*V* = 745.9 (3) Å^3^	Block, yellow
*Z* = 4	0.30 × 0.20 × 0.10 mm

### Data collection

**Table:**

Enraf–Nonius CAD-4 diffractometer	1063 reflections with *I* > 2σ(*I*)
Radiation source: fine-focus sealed tube	*R*_int_ = 0.039
Graphite monochromator	θ_max_ = 25.4°, θ_min_ = 2.7°
ω/2θ scans	*h* = 0→8
Absorption correction: ψ scan (North *et al.*, 1968)	*k* = −4→10
*T*_min_ = 0.962, *T*_max_ = 0.987	*l* = −13→13
2234 measured reflections	3 standard reflections every 200 reflections
1362 independent reflections	intensity decay: 1%

### Refinement

**Table:**

Refinement on *F*^2^	Secondary atom site location: difference Fourier map
Least-squares matrix: full	Hydrogen site location: inferred from neighbouring sites
*R*[*F*^2^ > 2σ(*F*^2^)] = 0.046	H-atom parameters constrained
*wR*(*F*^2^) = 0.146	*w* = 1/[σ^2^(*F*_o_^2^) + (0.1*P*)^2^ + 0.045*P*] where *P* = (*F*_o_^2^ + 2*F*_c_^2^)/3
*S* = 1.00	(Δ/σ)_max_ < 0.001
1362 reflections	Δρ_max_ = 0.22 e Å^−^^3^
119 parameters	Δρ_min_ = −0.20 e Å^−^^3^
0 restraints	Extinction correction: *SHELXL*, Fc^*^=kFc[1+0.001xFc^2^λ^3^/sin(2θ)]^-1/4^
Primary atom site location: structure-invariant direct methods	Extinction coefficient: 0.032 (7)

### Special details

**Table:**

Geometry. All esds (except the esd in the dihedral angle between two l.s. planes) are estimated using the full covariance matrix. The cell esds are taken into account individually in the estimation of esds in distances, angles and torsion angles; correlations between esds in cell parameters are only used when they are defined by crystal symmetry. An approximate (isotropic) treatment of cell esds is used for estimating esds involving l.s. planes.
Refinement. Refinement of F^2^ against ALL reflections. The weighted R-factor wR and goodness of fit S are based on F^2^, conventional R-factors R are based on F, with F set to zero for negative F^2^. The threshold expression of F^2^ > 2sigma(F^2^) is used only for calculating R-factors(gt) etc. and is not relevant to the choice of reflections for refinement. R-factors based on F^2^ are statistically about twice as large as those based on F, and R- factors based on ALL data will be even larger.

### Fractional atomic coordinates and isotropic or equivalent isotropic displacement parameters (Å^2^)

**Table:**

	*x*	*y*	*z*	*U*_iso_*/*U*_eq_	
N	0.8821 (2)	0.70812 (19)	1.15062 (16)	0.0546 (5)	
O2	0.6163 (2)	0.01034 (17)	0.87673 (13)	0.0722 (5)	
C2	0.6559 (3)	0.1292 (2)	0.91761 (18)	0.0539 (5)	
O1	0.62020 (18)	0.25817 (15)	0.85172 (12)	0.0545 (4)	
C8	0.6802 (2)	0.3797 (2)	0.91898 (15)	0.0433 (5)	
O3	0.9499 (2)	0.67114 (19)	1.24720 (15)	0.0811 (6)	
C9	0.7562 (2)	0.33732 (19)	1.02933 (14)	0.0404 (5)	
O4	0.8753 (3)	0.83771 (18)	1.11869 (17)	0.1003 (7)	
C3	0.7447 (3)	0.1713 (2)	1.03692 (16)	0.0535 (5)	
H3A	0.6724	0.1404	1.1015	0.064*	
H3B	0.8632	0.1266	1.0482	0.064*	
C7	0.6656 (3)	0.5261 (2)	0.88133 (16)	0.0510 (5)	
H7A	0.6133	0.5509	0.8068	0.061*	
C6	0.7322 (2)	0.6344 (2)	0.95957 (16)	0.0499 (5)	
H6A	0.7259	0.7352	0.9385	0.060*	
C5	0.8087 (2)	0.59213 (19)	1.06991 (15)	0.0426 (5)	
C4	0.8230 (2)	0.4451 (2)	1.10729 (15)	0.0417 (5)	
H4A	0.8755	0.4201	1.1818	0.050*	

### Atomic displacement parameters (Å^2^)

**Table:**

	*U*^11^	*U*^22^	*U*^33^	*U*^12^	*U*^13^	*U*^23^
N	0.0607 (10)	0.0423 (10)	0.0606 (11)	−0.0029 (7)	0.0005 (8)	−0.0091 (8)
O2	0.0933 (12)	0.0518 (10)	0.0695 (9)	−0.0181 (8)	−0.0122 (8)	−0.0149 (8)
C2	0.0595 (11)	0.0472 (11)	0.0541 (11)	−0.0113 (9)	−0.0035 (9)	−0.0039 (9)
O1	0.0621 (8)	0.0549 (9)	0.0445 (8)	−0.0074 (6)	−0.0134 (6)	−0.0041 (6)
C8	0.0433 (9)	0.0457 (11)	0.0401 (9)	−0.0042 (8)	−0.0045 (7)	−0.0030 (7)
O3	0.1034 (13)	0.0622 (10)	0.0728 (10)	−0.0035 (9)	−0.0338 (9)	−0.0167 (8)
C9	0.0400 (9)	0.0397 (9)	0.0405 (9)	−0.0032 (7)	−0.0051 (7)	0.0012 (7)
O4	0.170 (2)	0.0362 (9)	0.0916 (13)	−0.0105 (10)	−0.0194 (12)	−0.0042 (9)
C3	0.0669 (12)	0.0421 (10)	0.0495 (10)	−0.0091 (9)	−0.0121 (9)	0.0013 (8)
C7	0.0555 (11)	0.0544 (12)	0.0418 (9)	0.0021 (9)	−0.0089 (8)	0.0076 (8)
C6	0.0540 (11)	0.0416 (10)	0.0540 (11)	0.0044 (8)	0.0021 (8)	0.0069 (8)
C5	0.0394 (9)	0.0399 (10)	0.0483 (10)	0.0002 (7)	−0.0004 (7)	−0.0032 (8)
C4	0.0419 (9)	0.0421 (10)	0.0402 (9)	−0.0017 (7)	−0.0060 (7)	0.0005 (7)

### Geometric parameters (Å, º)

**Table:**

N—O4	1.210 (2)	C9—C3	1.485 (2)
N—O3	1.216 (2)	C3—H3A	0.9700
N—C5	1.460 (2)	C3—H3B	0.9700
O2—C2	1.185 (2)	C7—C6	1.379 (3)
C2—O1	1.385 (2)	C7—H7A	0.9300
C2—C3	1.508 (3)	C6—C5	1.387 (2)
O1—C8	1.380 (2)	C6—H6A	0.9300
C8—C7	1.374 (2)	C5—C4	1.378 (3)
C8—C9	1.385 (2)	C4—H4A	0.9300
C9—C4	1.374 (2)		
			
O4—N—O3	122.14 (18)	C9—C3—H3B	111.2
O4—N—C5	118.95 (17)	C2—C3—H3B	111.2
O3—N—C5	118.91 (17)	H3A—C3—H3B	109.1
O2—C2—O1	119.93 (17)	C8—C7—C6	116.70 (17)
O2—C2—C3	130.8 (2)	C8—C7—H7A	121.6
O1—C2—C3	109.23 (15)	C6—C7—H7A	121.6
C8—O1—C2	108.23 (13)	C7—C6—C5	119.61 (17)
C7—C8—O1	124.00 (15)	C7—C6—H6A	120.2
C7—C8—C9	123.75 (17)	C5—C6—H6A	120.2
O1—C8—C9	112.24 (15)	C4—C5—C6	123.47 (16)
C4—C9—C8	119.60 (16)	C4—C5—N	117.66 (16)
C4—C9—C3	132.86 (15)	C6—C5—N	118.85 (16)
C8—C9—C3	107.55 (15)	C9—C4—C5	116.86 (16)
C9—C3—C2	102.75 (15)	C9—C4—H4A	121.6
C9—C3—H3A	111.2	C5—C4—H4A	121.6
C2—C3—H3A	111.2		
			
O2—C2—O1—C8	−179.61 (18)	C9—C8—C7—C6	0.3 (3)
C3—C2—O1—C8	0.4 (2)	C8—C7—C6—C5	−0.1 (3)
C2—O1—C8—C7	−179.64 (16)	C7—C6—C5—C4	0.1 (3)
C2—O1—C8—C9	0.0 (2)	C7—C6—C5—N	178.69 (16)
C7—C8—C9—C4	−0.5 (3)	O4—N—C5—C4	178.15 (17)
O1—C8—C9—C4	179.89 (14)	O3—N—C5—C4	−1.0 (2)
C7—C8—C9—C3	179.23 (17)	O4—N—C5—C6	−0.5 (3)
O1—C8—C9—C3	−0.40 (19)	O3—N—C5—C6	−179.70 (17)
C4—C9—C3—C2	−179.74 (18)	C8—C9—C4—C5	0.4 (2)
C8—C9—C3—C2	0.61 (18)	C3—C9—C4—C5	−179.21 (17)
O2—C2—C3—C9	179.4 (2)	C6—C5—C4—C9	−0.2 (3)
O1—C2—C3—C9	−0.6 (2)	N—C5—C4—C9	−178.85 (14)
O1—C8—C7—C6	179.91 (16)		

### Hydrogen-bond geometry (Å, º)

**Table:**

*D*—H···*A*	*D*—H	H···*A*	*D*···*A*	*D*—H···*A*
C3—H3*A*···O2^i^	0.97	2.56	3.334 (3)	137

Symmetry code: (i) −*x*+1, −*y*, −*z*+2.
